# Nuts as functional foods: Variation of nutritional and phytochemical profiles and their *in vitro* bioactive properties

**DOI:** 10.1016/j.fochx.2022.100418

**Published:** 2022-08-08

**Authors:** Aneta Wojdyło, Igor Piotr Turkiewicz, Karolina Tkacz, Paulina Nowicka, Łukasz Bobak

**Affiliations:** a Department of Fruit, Vegetable and Nutraceutical Plant Technology, Wrocław University of Environmental and Life Sciences, 37 Chełmońskiego Street, 51-630 Wrocław, Poland; b Department of Functional Food Products Development, Wrocław University of Environmental and Life Sciences, 37 Chełmońskiego Street, 51-630 Wrocław, Poland

**Keywords:** Phenolics, Tocopherols, Tocotrienols, Triterpenes, Minerals, Sugars, PCA, Nuts, Food, Almonds, Brazil nuts, Cashews, Hazelnuts, Macadamias, Pecans, Pine nuts, Pistachios, Walnuts

## Abstract

The aim of the present study was to examine the nutritional (fat, fatty acids, minerals, sugars) and bioactive compounds (polyphenols, tocochromanols, triterpene) and their influence on *in vitro* anti-diabetic (pancreatic α-amylase and intestinal α-glucosidase), anti-obesity (pancreatic lipase) and anti-cholinergic (AChE and BuChE) inhibitory activity of 8 different popular nuts—pecan, pine, hazelnuts, pistachio, almonds, cashew, walnuts, and macadamia.

The total content of phenolic compounds in nuts ranged from 432.9 (walnuts) to 5.9 (pistachio) mg/100 g. The dominant polyphenols are polymeric procyanidins (walnuts – 415.1 mg/100 g). Nuts are rich in tocochromanols (832.9–4377.5 μg/100 g), especially α- and γ-tocopherols. The highest content of α-tocopherol (vitamin E) was detected in hazelnuts and almonds (2551.0 and 2489.7 μg/100 g, respectively) while the lowest amounts were detected in macadamia, cashew and walnuts. The most abundant in nuts are oleanic and pomolic acids (35 and 22 % of total, respectively), while betulin, uvaol and erythrodiol are less characteristic triterpenes for nuts (<1%). Pine nuts are the nuts with the highest content of triterpene (690.3 mg/100 g). Pistachio and almonds are the richest in total fat (>45 %), but monounsaturated (17.5–79.3 %; hazelnuts, almonds, cashew, macadamia, pistachio and pecan) and polyunsaturated (7.5–69.3 %; pine, walnuts) are the dominant fatty acids. Moreover, nuts are low in Cu, Zn, Mn and Na content, but rich in K (464.5–1772.3 mg/100 g) and Mg (197.0–502.5 mg/100 g). Macadamia, walnuts and cashew are good sources of Se. Pistachio, almonds and cashew were characterized by the highest content of sugars, but the dominant sugar was saccharose (58.2–2399.3 mg/100 g). All nuts showed high activity in inhibiting intestinal α-glucosidase (>90 %), but lower ability to inhibit pancreatic α-amylase and pancreatic lipase activity. The activity in inhibiting acetyl- and butylcholinesterase was up to 30 %.

As described above, all nuts contain various compounds that improve the nutritional value. Therefore they should be one of the important components of the daily human diet rich in functional foods.

## Highlights

• Nutritional, biological and *in vitro* anti-diabetic, -obesity, -cholinergic of nuts.
• Polymeric procyanidins dominant polyphenols.
• Oleanic and pomolic acids dominant triterpenes.
• Nuts are low in Cu, Zn, Mn, Na, but rich in K and Mg.
• All nuts showed high activity in inhibiting intestinal α-glucosidase.

## Introduction

Fruits, vegetable, herbs, grain, nuts and their products are consumed as typical foods or snacks of plant origin because they contain natural sources of health-promoting compounds. Currently among the most popular snack are edible nuts including almonds, Brazil nuts, cashews, hazelnuts, macadamias, pecans, pine nuts, pistachios, and walnuts due to their unique aroma, nutritional value, and health-related benefits. Nuts are a great example of a functional food because they contain bioactive compounds that support overall health, which has stimulated growing interest in them in the last decade (Alasalvar et al., 2020, Brufau et al., 2006, Cosmulescu et al., 2013). The health benefits of nuts are usually attributed to their chemical composition (Kim, Keogh, & Clifton, 2017). Nuts are typically sources of protein, dietary fiber, melatonin, fatty acids, plant sterols, vitamins such as folate, and tocopherols (Kim et al., 2017, Kornsteiner et al., 2006, Liu et al., 2014, Pino Ramos et al., 2019). In addition, nuts have other bioactive components such as polyphenols, especially polymeric procyanidins (Fregapane et al., 2020, Liu et al., 2014, Monagas et al., 2009, Pereira et al., 2008, Persic et al., 2018, Pino Ramos et al., 2019, Regueiro et al., 2014). Only a limited number of papers have described qualitative and quantitative triterpene compounds in fruits and leaves (Tkacz et al., 2019, Wojdyło et al., 2021, Wojdyło et al., 2021), but there are no literature data on their content in nuts. Triterpenes are secondary metabolites similar to polyphenols showing various and wide biological activities including antioxidant, antiallergic, antitumor, antiviral, anticancer, gastroprotective and neuroprotective and in multiple sclerosis drugs as well as anti- and pro-inflammatory cytokines (i.e. IL-1β, TNF-α and IL-6) (Agra et al., 2015, Grace et al., 2016, Sultana and Ata, 2008). The systematic consumption of nuts has been reported to decrease the risk of common civilization diseases (Kumawat et al., 2017, Rajaram and Sabaté, 2006, Ros and Mataix, 2006). Some research work (Ahola et al., 2021, Alasalvar et al., 2020, Kim et al., 2017) presents the position that the regular consumption of nuts is associated with an inverse risk for adiposity and metabolic syndrome contrary to the common claim that nuts contain a high level of fat (Kornsteiner et al., 2006). These results suggest that what is important is not only the content of fat, which is high, e.g. for macadamias, pecans, pines, Brazil nuts, walnuts approximately 76–64 % of dry matter (Kornsteiner et al., 2006), but primarily the type of fatty acid.

The chemical composition of nuts varies significantly according to the type of nuts, genotype, and differences between cultivars. Additionally, some researchers (Noguera-Artiaga et al., 2019, Persic et al., 2018) have noted that various factors such as agronomic practices and environmental conditions including temperature, rainfall, and exposure to light during growth, and finally maturity level at harvest and condition of the postharvest storage period have a significant influence on chemical composition and content of bioactive compounds. Therefore, it is important to know that nuts commonly purchased in commercial shops present a valuable range of nutritional compounds and are additionally rich in bioactive compounds.

Globally during 2020/21, over 5.3 million tonnes of tree nuts were produced and their production has increased considerably over the last few years. Currently it is predicted that the global market value of nuts is forecasted to reach about 1422.1 trillion U.S. dollars by 2023. Within the tree nut category, the almond production volume was the highest (1.6 million tonnes), followed by walnuts and cashews with over 1.1 million tonne production. The leading countries in terms of global nut production and export were China, USA, India, Turkey and Chile (STATISTA). Production increases year by year, alongside consumption, e.g. U.S. consumers ate approximately-five pounds of nuts in 2018.

Until now, there is only limited information about different nuts because when we buy some products on the label we can find only essential information about the content of fat, sugars, fiber and sodium. In the past years some researchers have focused on horticultural agronomy practice (Persic et al., 2018) or on basic chemical composition, e.g. fat, fatty acid (Kumawat et al., 2017, Ros and Mataix, 2006) and tocopherols (Pérez-Fernández et al., 2017) or polyphenols in walnuts (Beyhan, Gozlekci, Gundogdu, & Ercisli, 2016), hazelnuts (Pino Ramos et al., 2019), pistachio (Noguera-Artiaga et al., 2019;;Liu et al., 2014) or almonds (Bolling, Dolnikowski, Blumberg, & Chen, 2010). Increasingly, research is focused on waste nut products (Grace et al., 2016) and the importance of processing (Lainas et al., 2016, Monagas et al., 2009, Pelvan et al., 2018, Sonmezdag et al., 2019, Stuetz et al., 2017, Trox et al., 2010) or nut leaves (Amaral et al., 2004, Forino et al., 2016, Pereira et al., 2007), rather than on the nuts themselves.

Therefore, the aim of the present study was to examine the contents of nutritional (fat, fatty acids, minerals, sugars) and bioactive compounds (polyphenols, tocochromanols, triterpene) and their influence on *in vitro* anti-diabetic (pancreatic α-amylase and intestinal α-glucosidase), anti-obesity (pancreatic lipase) and anti-cholinergic (AChE and BuChE) inhibitory activity. Moreover, we analyzed the correlation between the inhibitory activity of enzymes and nutritional and bioactive compounds. The content of triterpene compounds was for the first time quantified in the analyzed nuts.

## Materials and methods

### 2.1 Standard compounds and chemicals

Standards from Extrasynthese (Genay, France): (−)-epicatechin, (+)-catechin, procyanidins B2, chlorogenic acid, cryptochlorogenic acid, quercetin-3-*O*-gulucoside—were used for the quantification of phenolic compounds. Hydrochloric acid, acetic acid, formic acid, ascorbic acid, acetonitrile, methanol, phloroglucinol, sodium acetate, sodium hydroxide, 3,5-dinitrosalicylic acid, potassium sodium tartrate tetrahydrate, sodium phosphate monobasic, starch from potato, pancreatic *α*-amylase from porcine pancreas (type VI-8), dipotassium hydrogen orthophosphate dihydrogen, *p*-nitrophenyl-α-d-glucopyranoside, and intestinal *α*-glucosidase from *Saccharomyces cerevisiae* (type I) were purchased from Sigma-Aldrich (Steinheim, Germany). The standard fatty acid methyl ester mix for quantification and Supelco 37 Component fatty acid methyl esters (FAME) mix were used.

### 2.2 Plant materials

Eight nuts (unknown variety name)—pecan (*Carya illinoinensis*), pine (*Pinus pinea*), hazelnuts (*Corylus avellana*), pistachio (*Pistacia vera*), almonds (*Prunus amygdalus*), cashew (*Anacardium occidentale L.*), walnuts (*Juglans regia),* macadamia (*Macadamia integrifolia*)—were obtained from a commercial shop on November 2021. All analyzed nuts were natural and no salted but nuts as pine, almonds, cashew and macadamia were without skin. Additionally, all kinds of nuts were purchased from the company Lidl (Wrocław, Poland), which possesses in its portfolio different types of nuts and dried fruits for the Poland market. Approximately 0.3 kg of each sample was subsequently ground in an analytical mill (IKA 11A basic; Staufen, Germany) for analysis.

### 2.3 Analysis of sugars

Sugars from nuts were analyzed using an high -performance liquid chromatography cooperated with evaporated light scattering detector (Merck, Hitachi, Japan) by the method as described previously (Tkacz et al., 2019, Wojdyło et al., 2021). All measurements were performed in n = 3 and expressed as mg per 100 g of nuts.

### 2.4 Analysis of minerals

The mineral content was analyzed using wet washed nuts by methods described previously (Tkacz et al., 2021, Wojdyło et al., 2021). Selected minerals (Na, K, Mg, Ca, Fe, Cu, Zn, Mn and Se) were analyzed using an atomic absorption spectrophotometer model AA-7000 series (SHIMADZU CORP.; Kyoto, Japan). Standard calibration curves (R^2^ ≥ 0.9990) were constructed for all minerals based on the reference standard provided by AccuStandard (New Haven, CT, USA). All incubations were measurements in n = 3 and expressed as mg per 100 g of nuts, except selenium, which was expressed as μg per 100 g of nuts.

### 2.5 Analysis of polyphenols

For the determination of polyphenols samples (〜0.3 g) homogenized with solvents as ascorbic acid/ methanol/water hexane (1:29:30:30, m/*v*/*v/v*) was mixed intensively according to the guidelines previously described (Pérez-Fernández et al., 2017, Tkacz et al., 2021, Wojdyło et al., 2021). Next, it was subjected to an extraction process in a ultrasonic bath (PX 40A, CNC-Ultrasonic.pl; Jedlina Letnisko, Poland) for 15 min at 25 °C and left for 15 h at 4 °C. After this time, the samples were re-extracted for 15 min and centrifuged at 14,000*g* for 15 min at 20 °C (M−Universal, MPW Med. Instruments; Warsaw, Poland). Finally, the supernatant was filtered through 0.20 µm hydrophilic PTFE filters (Millipore Millex Samplicity, Merck; Darmstadt, Germany) before analysis.

Polyphenols from nuts were analyzed using an Acquity UPLC system equipped with a photodiode and fluorescence detector with binary solvent manager (Waters Corp., Milford, MA, USA) series according to the guidelines of Wojdyło, Nowicka, Turkiewicz, and Tkacz (2021). Before injection the supernatant was filtered through 0.20 µm hydrophilic PTFE filters (Millipore Millex Samplicity, Merck; Darmstadt, Germany). The prepared extract mixture was separated in a BEH C18 column (2.1 × 100 mm, 1.7 μm). The 2 % an aqueous solution of formic acid (v/v, solvent A) and acetonitrile (solvent B) used as following gradient program: initial conditions, 99 % (A); at 12.00 min, 65 % (A); at 12.50 min, 100 % (B); and, at 13.50 min, 99 % (A). Quantification was achieved by standard compounds as (−)-epicatechin, (+)-catechin, procyanidin B2, chlorogenic, neochlorogenic acids, and quercetin-3-*O*-glucoside after prepared calibration curves from 0.5 to 5.0 mg/mL (R^2^ ≥ 0.997 – 0.999).

Analysis of polymeric procyanidins was performed by the phloroglucinolysis method according to guidelines (Turkiewicz et al., 2021, Wojdyło et al., 2021) using a UPLC-FL Acquity system. The mixture was separated in a BEH C18 RP column (2.1 × 5 mm, 1.7 μm). The 2.5 % an aqueous solution of acetic acid (v/v, solvent A) and acetonitrile (solvent B) used as following gradient program: 0–1.00 min, 2 % B; 1.00–2.50 min, 2–3 % B; 2.50–3.50 min, 3–10 % B; 3.50–5.50 min, 10–15 % B; 5.50–7.50 min, 100 % B; finally, washing and reconditioning of the column for next 2.50 min. The data acquisition and processing was carried out using by Empower 2.0 Manager software. All samples were measurements in n = 3 and the results were expressed as mg per kg.

### 2.6 Analysis of triterpenes

About 0.3 g of the nuts was weighed and extracted by method previously guidelines (Turkiewicz et al., 2021, Wojdyło et al., 2021). Analysis was performer using a UPLC-PDA Acquity system. The mixture was separated in a ZORBAX Eclipse PAH column (3.5 µm, 2.1 mm × 150 mm) protected by a guard column and were monitored at λ = 210 nm at flow rate of 0.25 mL/min. The mobile phase consisted water (solvent A) and acetonitrile (solvent B) used as following gradient program: 45–10 % solvent A (0–15.0 min), and then lowering solvent B from 90 to 55 % (15.1–20.0 min) to the initial composition for held constant to re-equilibrate the column. Quantification was achieved by standard compounds after prepared calibration curves from 10 to 380 mg/L (R^2^ ≥ 0.997 – 0.999). All samples were done n = 3 and the results were expressed as mg per 100 g of nuts.

### 2.7 Analysis of tocochromanols

About 1 g of the nuts was weighed and extracted by the method previously guidelines (Turkiewicz et al., 2021, Wojdyło et al., 2021) using a UPLC-FL Acquity system (Waters Corp., Waters Corp.; Ireland). The mixture was separated in a BEH Shield RP18 column (1.7 µm, 2.1 mm × 100 mm), protected by a guard column and were monitored at excitation and emission at λ = 290 and 330 nm, respectively, at flow rate of 0.45 mL/min. An methanol (solvent A) and 0.1 % aqueous solution of formic acid (v/v, solvent B) in the proportion 88:12 (v/v) were used for isocratic elution. Calibration curves (R^2^ ≥ 0.9990) were prepared from α-, β-, γ-, δ-tocopherol and -tocotrienol in the concentration range from 17 to 500 mg/L. All samples were done n = 3 and the results were expressed as μg per 100 g of nuts.

### 2.8 Analysis of fatty acids

Nut samples were derivatized for analysis of fatty acids as guidelines (Kornsteiner et al., 2006, Tkacz et al., 2019) using a gas chromatograph equipped with a quadrupole mass detector (Agilent Technologies Inc., Santa Clara, CA, US). The mixture was separated in an HP-88 capillary column (0.25 mm × 100 m) filled with a cyanopropylaryl polysiloxane bed (88:12) with grain size of 0.2 μm and used for analysis. The results of fatty acid studies were expressed as the percentage of total fatty acids of nuts.

### *2.9 In vitro* anti-diabetic, -obesity and -cholinergic activity

For the determination of *in vitro* inhibitory activity methanol/water/hydrochloric acid (80:19:1, *v/v/v*) as a solvent was used for extraction as guidelines in the literature (Pérez-Fernández et al., 2017, Tkacz et al., 2021, Wojdyło et al., 2021).

The activity of pancreatic α-amylase, intestinal α-glucosidase and pancreatic lipase were determined as guidelines in the literature (Pino Ramos et al., 2019, Tkacz et al., 2019, Wojdyło et al., 2021) by colorimetric assay at 600, 405, and 400 nm, after 10, 10, and 15 min at 37 °C incubation, respectively. Results are expressed as the amount of sample that is able to inhibit by 50 % (IC_50_, mg/mL) and % of inhibition. Orlistat (1 mg/mL) and acarbose (1 mg/mL) were used as the reference compounds.

The inhibitory activities of acetylcholinesterase (AChE) and butylcholinesterase (BuChE) were guidelines in the literature by colorimetric assay at 412 nm after 3 min of reaction. Galantamine (1 mg/mL) was used as a reference compound. Results are expressed as % of inhibition.

All inhibitory assays were performed in triplicate using a Synergy H1 microplate reader (BioTek, Winooski, VT, US).

### 2.10 Statistical analysis

The values presented in tables are refer means ± standard error of the mean (SEM). Statistical analysis was conducted using *XLSTAT* 2021 software version (Addinsoft, New York, NY, USA). Principal components analysis (PCA) was performed to identify relationship and additionally Person’s correlations was calculated between bioactive components and biologically active properties. One-way analysis of variance (ANOVA) by Tukey’s test was used for statistically significant differences analyzed at p ≤ 0.05.

## Results and discussion

### Polyphenols, tocochromanols and triterpene in nuts

**Fig. 1:**
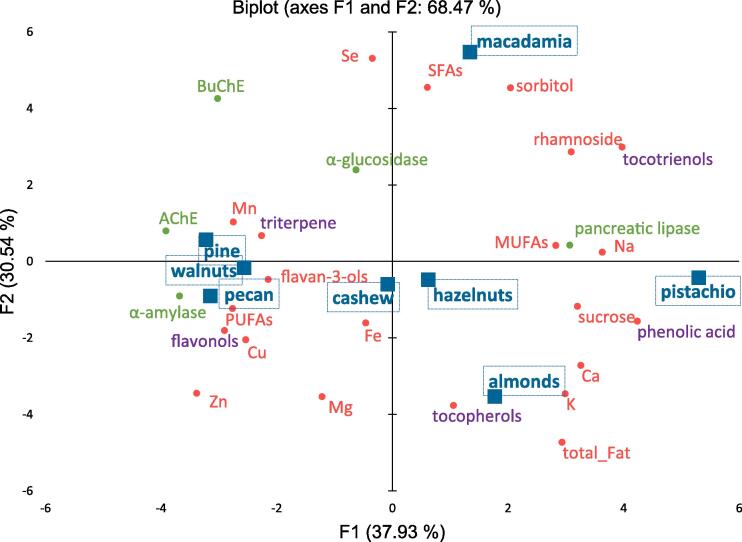
Correlation circles of principal components (PCA) in two axis. SFAs – saturated fatty acids; MUFAs - monounsaturated fatty acids; PUFAs - polyunsaturated fatty acids; AChE – acetylocholinesterase; BuChE – butylocholinesterse.

*Polyphenols*. Among the polyphenols, phenolic acids, flavonols and polymeric procyanidins were quantified in tested nuts and the results are summarized in Table 1. The total content of phenolic compounds in nuts ranged from 432.9 mg (walnuts) to 5.9 mg (pistachio) in 100 g. In pecan, hazelnuts, almonds and cashew the total polyphenol content was lower than 100 mg/100 g, while in pine and macadamia nuts polyphenols were not detected. The dominant polyphenols in nuts are polymeric procyanidins belonging to flavanols, i.e. in walnuts – 415.1 mg, cashew – 68.3 mg, pecan – 52.4 mg/100 g. Phenolic acid were quantified only in pistachio and almonds, whereas flavonols were quantified only in pecan, walnuts, and almonds. Flavanols and flavonols positively associated with walnuts, pecan and cashew had a stronger influence on pancreatic α-amylase as revealed by PCA analysis (Fig. 1). Phenolic acid characteristics for almonds and pistachio negatively influenced intestinal α-glucosidase, AChE and BuChE activity.

**Table 1:** Quantification of phenolic compounds, tocochromanols (tocopherols and tocotrienols), triterpene, fat and fatty acids (SFAs, MUFAs, PUFAs), minerals, sugars and *in vitro* activity (pancreatic- α-amylase, intestinal α-glucosidase, AChE and BuChE) composition in different nuts.

	Nuts							
	pecan	pine	hazelnuts	pistachio	almonds	cashew	walnuts	macadamia
*Polyphenols [mg/100 g]*								
Flavanols	52.4 ± 2.4c	nd	12.0 ± 0.6d	5.0 ± 0.4de	8.8 ± 1.1de	68.3 ± 1.5b	415.1 ± 2.7a	nd
Phenolic acids	nd	nd	nd	0.9 ± 0.1a	0.3 ± 0.0b	nd	nd	nd
Flavonols	23.5 ± 1.7a	nd	nd	nd	7.1 ± 0.5c	nd	17.8 ± 1.1b	nd
Σ *Polyphenols*	75.9b	nd	12.0c	5.9d	16.3c	68.3b	432.9a	nd

*Tocochromanols [*μg/100 g]								
δ-Tocotrienols	5.3 ± 0.3c	2.3 ± 0.2c	2.1 ± 0.1c	238.2 ± 3.7a	2.5 ± 0.3c	66.3 ± 2.8b	2.3 ± 0.3c	1.3 ± 0.2c
β-Tocotrienols	4.7 ± 0.1b	2.3 ± 0.1b	6.8 ± 0.3b	664.3 ± 5.8a	1.9 ± 0.1b	nd	nd	3.0 ± 0.1b
γ-Tocotrienols	10.5 ± 0.8e	157.1 ± 1.6a	19.8 ± 1.4d	139.7 ± 1.6b	4.6 ± 0.4f	36.0 ± 3.5c	17.3 ± 0.7d	16.9 ± 0.5d
α-Tocotrienols	30.6 ± 1.4de	8.7 ± 0.7e	32.5 ± 1.3d	164.2 ± 3.1b	113.2 ± 3.8c	14.4 ± 1.1de	33.7 ± 1.5d	864.1 ± 3.1a
Σ Tocotrienols	51.1e	170.4c	61.1e	1206.4a	122.2d	116.6d	53.3e	885.2b
δ-Tocopherols	171.7 ± 4.3b	31.0 ± 2.4d	8.6 ± 0.5e	78.5 ± 1.2c	7.5 ± 0.4e	38.3 ± 1.1d	220.9 ± 4.1a	72.6 ± 4.2c
β-Tocopherols	7.5 ± 1.4e	29.8 ± 1.2d	296.4 ± 1.5a	42.1 ± 0.7c	120.5 ± 3.5b	2.0 ± 0.2e	nd	0.3 ± 0.0e
γ-Tocopherols	2575.8 ± 2.9b	711.4 ± 6.4d	151.8 ± 10.1f	2855.2 ± 2.7a	70.8 ± 1.3f	612.9 ± 3.7de	1611.5 ± 7.9c	571.5 ± 2.7e
α-Tocopherols*	150.4 ± 4.2 cd	1118.2 ± 8.5b	2551.0 ± 4.8a	195.2 ± 3.1c	2489.7 ± 4.7a	63.1 ± 1.1de	76.7 ± 2.0de	27.9 ± 2.5e
Σ Tocopherols	2905.4 ± 6.8e	1890.3 ± 5.2c	3007.7 ± 5.2e	3171.1 ± 5.7a	2688.6 ± 4.6d	716.3 ± 2.7d	1909.1 ± 5.9e	672.3 ± 5.2b
Σ T *ocochromanols*	2956.5b	2060.7d	3068.9ab	4377.5a	2810.8c	832.9e	1962.4d	1557.5e

*Triterpene mg/100 g[]*								
Tormentic acid	32.7 ± 0.7b	20.6 ± 1.0d	18.3 ± 2.1e	23.2 ± 1.3c	5.6 ± 0.3 g	14.1 ± 1.5f	14.9 ± 1.1f	40.4 ± 1.7a
Maslinic acid	13.9 ± 0.3a	12.2 ± 0.4b	10.8 ± 1.1 cd	11.1 ± 0.8b	6.4 ± 0.5e	10.2 ± 1.2d	10.4 ± 0.9 cd	12.3 ± 0.7b
Pomolic acid	79.6 ± 1.4a	71.0 ± 1.3bc	66.5 ± 2.6 cd	62.9 ± 3.1de	35.5 ± 1.4 g	60.2 ± 1.7ef	57.9 ± 1.5f	74.5 ± 2.5b
Corosolic acid	23.6 ± 0.6e	27.4 ± 0.4bc	25.8 ± 3.1 cd	18.4 ± 1.4f	30.0 ± 1.4a	26.4 ± 1.4bc	24.3 ± 2.6de	28.0 ± 1.1b
Betulinic acid	1.0 ± 0.0c	24.6 ± 1.1a	1.8 ± 0.5c	4.1 ± 0.2b	1.2 ± 0.1c	1.3 ± 0.3c	24.4 ± 1.5a	0.1 ± 0.0d
Oleanolic acid	15.7 ± 0.5e	378.0 ± 2.5a	39.1 ± 1.4 cd	47.2 ± 2.6c	29.4 ± 1.3d	30.9 ± 0.9d	60.3 ± 1.7b	5.3 ± 0.5f
Ursolic acid	1.7 ± 0.2f	27.2 ± 0.4a	7.2 ± 0.7c	2.5 ± 0.9e	3.4 ± 0.2d	11.9 ± 1.5b	3.2 ± 1.6de	2.5 ± 0.2ef
Betulin	0.5 ± 0.1b	0.2 ± 0.0d	nd	0.4 ± 0.1c	1.0 ± 0.1a	nd	0.2 ± 0.0e	nd
Erythrodiol	nd	0.2 ± 0.0c	nd	nd	1.2 ± 0.2a	nd	0.4 ± 0.0b	nd
α-Boswellic acid	0.8 ± 0.1f	118.8 ± 2.1a	83.4 ± 2.5b	30.9 ± 2.1c	19.8 ± 1.1d	28.8 ± 2.6c	2.9 ± 0.6ef	5.3 ± 0.5e
Uvaol	0.2 ± 0.0de	10.0 ± 1.1a	1.1 ± 0.2c	1.4 ± 0.3b	0.3 ± 0.0d	nd	0.2 ± 0.0de	0.4 ± 0.1d
Σ *Triterpene*	169.8d	690.3a	253.9b	202.1c	133.9e	183.7 cd	199.2c	168.9d

Fat and fatty acid [%]								
Σ Fat	34.1 ± 1.3c	34.5 ± 2.4bc	35.3 ± 1.5bc	46.7 ± 2.6a	45.5 ± 2.1a	36.8 ± 2.5b	30.8 ± 1.5d	24.9 ± 1.2e
SFAs	12.6 ± 0.4 cd	11.8 ± 1.1d	13.3 ± 0.7c	13.3 ± 1.1c	9.2 ± 1.1e	20.3 ± 2.4b	13.3 ± 1.3c	21.5 ± 1.4a
MUFAs	57.2 ± 1.1 cd	32.2 ± 0.6e	79.3 ± 1.1a	55.4 ± 2.5d	72.7 ± 2.4b	61.4 ± 2.7c	17.5 ± 0.9f	75.6 ± 2.5ab
PUFAs	30.2 ± 0.5c	56.0 ± 1.4b	7.5 ± 0.6e	31.3 ± 1.5c	18.1 ± 2.1d	18.4 ± 1.1d	69.3 ± 2.4a	2.8 ± 0.6f

*Minerals [mg/100 g]*								
sodium (Na)	4.4 ± 0.3e	5.3 ± 0.6e	5.4 ± 0.2e	55.6 ± 2.5a	8.4 ± 1.1d	45.3 ± 3.3b	4.9 ± 0.5e	17.9 ± 1.6c
potassium (K)	718.7 ± 5.2f	1233.6 ± 6.2d	1627.2 ± 2.9b	1772.3 ± 5.2a	1511.1 ± 4.7c	958.1 ± 5.8e	629.5 ± 2.7 g	464.5 ± 2.4 h
magnesium (Mg)	296.3 ± 6.6c	485.6 ± 3.7a	348.7 ± 2.7b	251.9 ± 2.7d	482.8 ± 4.4a	502.5 ± 3.6a	271.5 ± 3.1 cd	197.0 ± 1.5e
calcium (Ca)	19.2 ± 0.4e	1.5 ± 0.1 g	36.6 ± 1.5c	41.8 ± 1.5b	59.0 ± 2.7a	14.9 ± 2.1f	34.4 ± 1.6c	24.6 ± 1.1d
iron (Fe)	8.1 ± 1.1e	11.7 ± 0.4b	8.8 ± 0.7de	10.0 ± 0.9c	11.2 ± 1.1b	14.0 ± 1.3a	11.3 ± 1.7b	9.0 ± 0.4d
copper (Cu)	1.3 ± 0.2d	1.6 ± 0.1c	1.8 ± 0.1b	0.8 ± 0.1f	0.9 ± 0.1e	2.5 ± 0.4a	1.8 ± 0.3b	0.5 ± 0.0 g
zinc (Zn)	3.3 ± 0.2a	3.3 ± 0.2a	1.8 ± 0.3d	1.8 ± 0.5d	2.3 ± 0.3c	3.3 ± 0.2a	2.5 ± 0.4b	0.7 ± 0.1e
manganese (Mn)	1.5 ± 0.4e	7.5 ± 0.5a	5.5 ± 0.6b	0.9 ± 0.2f	1.7 ± 0.2e	1.6 ± 0.3e	4.0 ± 0.2c	2.6 ± 0.3d
selenium (Se) [μg/100 g]	0.6 ± 0.1f	3.5 ± 0.4d	nd	1.4 ± 0.3e	nd	5.0 ± 0.4c	6.8 ± 0.5b	13.1 ± 1.3a
Σ *Minerals*	1052.9f	1750.0c	2035.7b	2135.0a	2077.5e	1542.2e	959.9d	716.8b

*Sugars [mg/100 g]*								
Rhamnose	nd	19.6 ± 1.8c	26.3 ± 1.9b	37.7 ± 1.7a	6.4 ± 0.6e	5.9 ± 0.3e	17.7 ± 1.1d	27.6 ± 1.7b
Sorbitol	nd	52.8 ± 2.5c	33.7 ± 2.9d	56.2 ± 3.2b	nd	nd	nd	76.4 ± 2.6a
Sucrose	1785.6 ± 5.2c	1308.9 ± 4.7d	950.5 ± 5.2e	2399.3 ± 7.5a	2153.6 ± 5.5b	2140.5 ± 3.8b	888.3 ± 7.5e	1711.9 ± 8.3c
Σ *Sugars*	1785.6c	1381.3d	1010.5e	2493.2a	2160.0b	2146.4b	906.0e	1815.9c

*In vitro activity*								
Pancreatic α-amylase [IC_50_]	148.9 ± 2.6a	262.6 ± 3.1b	276.5 ± 2.6c	391.0 ± 1.3e	262.1 ± 3.4c	318.9 ± 3.3e	281.0 ± 2.4c	284.1 ± 2.8d
Pancreatic α-amylase [%]	60.7 ± 2.6a	43.1 ± 3.1b	37.0 ± 2.6c	26.8 ± 1.3e	35.5 ± 3.4c	29.0 ± 3.3e	35.4 ± 2.4c	32.5 ± 2.8d
Intestinal α-glucosidase [IC_50_]	13.9 ± 1.1a	16.1 ± 1.6a	17.5 ± 1.3a	17.6 ± 2.1a	18.5 ± 1.6a	17.7 ± 1.3a	17.1 ± 2.5a	15.2 ± 0.6a
Intestinal α-glucosidase [%]	99.2 ± 1.1a	99.9 ± 1.6a	98.0 ± 1.3a	98.0 ± 2.1a	93.4 ± 1.6a	98.4 ± 1.3a	93.4 ± 2.5a	98.1 ± 0.6a
Pancreatic lipase [%]	127.2 ± 3.2 g	38.5 ± 1.4ef	49.5 ± 2.5c	68.8 ± 2.6a	36.3 ± 1.4f	41.2 ± 2.6de	57.1 ± 2.1b	42.2 ± 1.9d
AChE [%]	29.1 ± 1.3a	27.3 ± 1.1b	13.5 ± 0.9de	12.7 ± 0.5e	8.1 ± 0.9 g	11.3 ± 0.6f	17.1 ± 1.1c	14.1 ± 1.3d
BuChE [%]	10.1 ± 0.5b	10.6 ± 0.9b	1.9 ± 0.2d	1.8 ± 0.1d	nd	1.1 ± 0.1e	7.3 ± 0.4c	12.2 ± 0.5a

†significant at p < 0.05; ‡Values (mean of 3 replications) followed by the same letter, within the same row were not significantly different (p < 0.05), according to the Tukey's least significant difference test.

n.d. – not detected; TT- tocotrienols; TF- tocopherols; vitamin E = α-tocopherols; SFAs – saturated fatty acids; MUFAs - monounsaturated fatty acids; PUFAs - polyunsaturated fatty acids; AChE – acetylocholinesterase; BuChE – butylocholinesterse; IC_50_ - mg/mL;

As described in the literature, total polyphenol content in nuts is 34–2052 mg/100 g (Bolling et al., 2010, Monagas et al., 2009). Polyphenols in nuts are mainly located in the testa or skin, which is usually removed during the blanching or roasting process (Monagas et al., 2009). Rodríguez-Bencomo et al. (2015) elucidated that the total phenolic content increased alongside the heat treatment in the pistachio samples, with the double-roasted nuts having the highest total phenolic content (42.4 mg/100 g fw) compared to the single-roasted and raw (32.4 and 26.2 mg/100 g fw, respectively) samples. Pistachio, pecan, hazelnuts, almonds or cashew, pine, and macadamia are typically commercial nuts without skin; therefore, this fact partially explains the low content of polyphenols. Bolling et al. (2010) reported that the content of polyphenols for different almond cultivars ranged between 4.0 and 10.7 mg/100 g but Bittner, Rzeppa, and Humpf (2013) mentioned 148 μg/100 g in macadamia nuts. Beyhan et al. (2016) reported total polyphenols of walnuts in different genotypes ranging from 1107 to 1876 mg gallic acid (GAE)/100 g. Wu et al. (2004) reported total phenol contents ranging from 68 to 1556 mg GAE/100 g for 10 different nuts, but pine and walnut were characterized by the lowest and highest content, respectively. Monagas et al. (2009), and Gu et al. (2004), reported that flavanols such as polymeric procyanidins, especially polymeric procyanidins, are the most abundant polyphenols fraction in nuts. Recent studies (Beyhan et al., 2016, Wu et al., 2021) demonstrated that walnuts had the highest content of polymeric procyanidins, which are responsible for the astringent taste and color of skins. It is known that polymeric procyanidins are of great interest as important compounds in nutrition and biological activity as they exhibit antioxidant, anti-inflammatory, antimicrobial, cardio- and neuroprotective action (Monagas et al., 2009, Wang et al., 2011). Gu et al., 2004, Wang et al., 2011 analyzed the concentrations of polymeric procyanidins in different food samples and estimated the average daily intake as about 57.7 and 95.0 mg/day/person, respectively. However, today, tea, red wine, cocoa or chokeberry fruits but not nuts still are major contributors to intake of polymeric procyanidins in the diet in different countries.

**Fig. 2:**
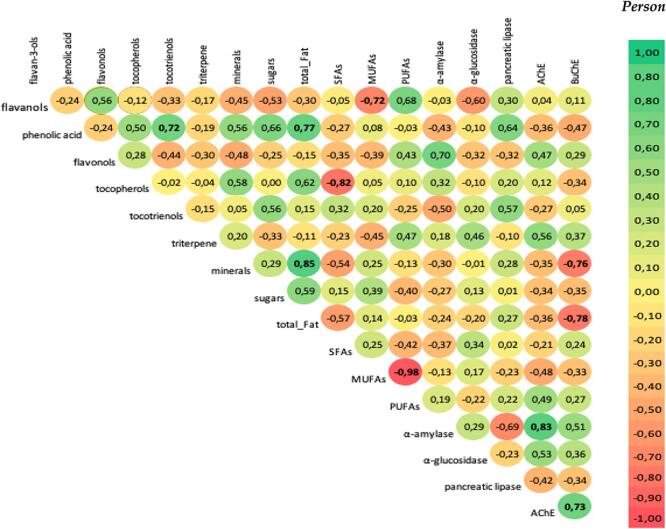
Pearson's correlation coefficient showing the strength of relationship between variables.Values in bold are significant at 95 % confidence limit.

*Tocochromanols.* The lipophilic fraction of nuts contains tocopherols and tocotrienols. The highest content of total tocochromanols was found in pistachio (4377.5 μg), hazelnuts (3068.9 μg) pecan (2956.5 μg) and almonds (2810.8 μg) while cashew nuts had the poorest content (832.9 μg) per 100 g. The tocopherol isomers can be ranked by decreasing content: γ > α > δ > β, averaging 57.2, 34.8, 5.5 and 2.5 % of total tocopherols. Among tocotrienols the relationship was α > β > γ > δ, averaging 47.7, 24.9, 15.7 and 11.8 % of total tocotrienols. However, the greatest pro-health potential is attributed to vitamin E as the equivalent of α-tocopherol content, which presents the highest biological activity. Therefore, the highest content of α-tocopherol (vitamin E) was detected in hazelnuts and almonds (2551.0 and 2489.7 μg/100 g, respectively) whiles the lowest amounts were detected in macadamia, cashew and walnuts (27.9, 63.1, and 76.7 μg/100 g, respectively). γ-Tocopherols dominated in pistachio and pecan. The highest content of tocotrienols was measured in pistachio (1206.4 μg/100 g). Additionally, α-tocotrienol (55.5 %) was predominant isomers of tocochromanols in macadamia nuts. Tocopherols which present high content in pistachio, almonds and hazelnuts were weakly associated in an *in vitro* test evaluated in this work with antidiabetic, anti-obesity and anticholinergic activity. However, tocotrienols present in macadamia nuts positively were associated with pancreatic lipase (Fig. 1, Fig. 2).

The analyzed results are in line with previous data (Beyhan et al., 2016, Kornsteiner et al., 2006, Kumawat et al., 2017, Stuetz et al., 2017). Experimental investigations by Stuetz et al. (2017) revealed that almonds and hazelnuts were abundant in α-tocopherol while walnuts were rich in γ-tocopherol. Pistachios are a very good source of γ-tocopherols with an average level of around 30 mg/100 g (Stuetz et al., 2017), but in different varieties of pecan nuts vitamin E content ranged from 9 to 32 mg/100 g (Pérez-Fernández et al., 2017). Kornsteiner et al. (2006) analyzed tocopherols (but did not evaluate tocotrienols) from extracted oil in 10 different popular nuts (almonds, cashews, hazelnuts, macadamias, peanuts, pecans, pines, pistachios and walnuts). They reported that the mean maximum content of α-tocopherol ranged from 33.1 mg/100 g for hazelnuts to a non-detectable content for macadamias nuts. Among all nuts, almonds and hazelnuts had the highest mean α-tocopherol content (24.2 and 31.4 mg/100 g extracted oil, respectively). In the rest of the nuts, the content of β- and γ-tocopherols was between 5.1 and 29.3 and δ-tocopherol below < 4 mg/100 g of extracted oil (Kornsteiner et al., 2006). The low content of tocopherols in some nuts probably results from the nature of tocopherol, because these compounds are sensitive to degradation when exposed to oxygen and light (Trox et al., 2010).

The recommended daily allowance (RDA) intake of vitamin E (as α-TE) for adults women and men is set at 11 and 13 mg/day, respectively. The content of vitamin E (Table 1) shows that some nuts are excellent sources of this compound and they can contribute to a balanced daily intake of vitamin E. Nevertheless, the average intake of nuts is about 4 g/day, which is still insignificant for observing a beneficial influence of dietary bioactive components of nuts in the diet. Therefore the proposition is to increase the daily intake of nuts to 20–30 g/day, especially hazelnuts and almonds. In reference to RDA, low levels of vitamin E in the diet could be related to a potentially higher risk of atherosclerosis or degenerative diseases and regular intake appears to be protective against these diseases (Kornsteiner et al., 2006). Nonetheless, even low but regular consumption of nuts has been shown to have beneficial effects on cardiovascular diseases (Bramley, Elmadfa, Kafatos, Kelly, Manios, Roxboroug, & Schuch, 2000).

*Triterpene.* Eleven triterpene compounds belonging to ursanes, oleananes and lupanes were identified and quantified in nuts (Table 1). It is worth noting that this is the first study to quantify triterpene compounds in nuts. The compounds have attracted attention due to their remarkable biological activities (Agra et al., 2015, Grace et al., 2016, Sultana and Ata, 2008).

The most abundant triterpene compounds in nuts are oleanic and pomolic acid (mean of total triterpene 35 and 22 %, respectively), while betulin, uvaol and erythrodiol are less characteristic triterpenes for nuts (mean of total triterpene < 1 %). Pine nuts are the nuts with the highest content of triterpene (690.3 mg/100 g) and oleanolic acid amounted to almost 50 % of total triterpene. Triterpene content in pine had a strong influence on intestinal α-glucosidase and AChE and BuChE activity (Fig. 1, Fig. 2). The rest of the nuts contain triterpene at levels from 168.9 (pecan and macadamia) to 253.9 mg (hazelnuts) per 100 g of nuts. Compared to other plant sources the analyzed nuts exhibit higher content of triterpene compared to sea buckthorn berries (Tkacz et al., 2021), flowering quince seeds (Turkiewicz et al., 2021) or fruits and leaves of apple, pears or quinces (Wojdyło, Nowicka, Turkiewicz, Tkacz, et al., 2021).

It is known that many triterpenoids present antimicrobial, anti-cancer, chemopreventive, hepatoprotective, anti-inflammatory, and antioxidant activities (Grace et al., 2016, Tkacz et al., 2021). Oleanolic acid has been used as a therapeutic agent with gastroprotective and cardioprotective action, in models of diabetes to improve insulin action, and in the treatment of allergic asthma and liver disorders, and shows an immunomodulatory effect (Agra et al., 2015, Grace et al., 2016, Sultana and Ata, 2008). Pomolic acid diminished mean arterial blood pressure used in the prevention of cardiovascular diseases and has anti-carcinogenic (Agra et al., 2015) and anti-HIV properties (Sultana & Ata, 2008).

### Fatty acids, minerals and sugars in nuts

*Fatty acids.* The total fat and fatty acid content in nuts is summarized in Table 1. Pistachio and almonds are the nuts that are richest in total fat (>45 %) whereas the remaining nuts contain fats in the range 30.8–36.8 %, but macadamia nuts content the lowest value, >25 % fats. The content of total fat and fatty acid in nuts is similar to values previously presented in the literature (Brufau et al., 2006, Pereira et al., 2008), but slightly lower compared to results reported by other authors (Kornsteiner et al., 2006, Ros and Mataix, 2006). The differences between results primarily follow from variance in species, environmental factors and different methodologies used for analysis.

Nevertheless, nuts are sources of high energy (9 kcal/g compared with 4 kcal/g for carbohydrate and protein) (Brufau et al., 2006, Ros and Mataix, 2006) due to their total fat content. Therefore, nuts during the last decades have been placed with care in the food pyramid. Notwithstanding, this point of view has now been reconsidered and nowadays nuts are recommended for daily consumption as beneficial effects were considered and proven for monounsaturated fatty acids. Nevertheless, to integrate nuts in a healthy diet a daily intake of 25 g is recommended (Ahola et al., 2021).

Three types of fatty acids were identified in the studied nuts: saturated (9.2–20.3 %; SFAs), monounsaturated (17.5–79.3 %; MUFAs) and polyunsaturated (7.5–69.3 %; PUFAs) fatty acids (Table 1). The dominant fatty acid was monounsaturated type (hazelnuts, almonds, cashew, macadamia, pistachio and pecan) and the nuts were also abundant in polyunsaturated fatty acids (pine, walnuts). Many experimental investigations reported (Kornsteiner et al., 2006, Kumawat et al., 2017, Pereira et al., 2008, Ros and Mataix, 2006) that among the different nuts macadamia is low, hazelnuts and cashews are moderately low, almond and pistachios are moderately high, pecans are high, and walnut and pine nuts are very high in polyunsaturated fatty acid content. Walnuts are good sources of fatty acids, especially omega-3 and omega-6, as linoleic followed by oleic, linolenic, palmitic and stearic acid as polyunsaturated fatty acids have been associated with beneficial effects on serum lipids (Beyhan et al., 2016). PUFAs of walnuts and pecan and all nuts’ content of MUFAs had a positive influence on pancreatic lipase inhibitory (Fig. 1).

The high content of monounsaturated fatty acid has a good affect on health, i.e. it can help reduce bad cholesterol (HDL) levels in blood, which can lower the risk of heart disease, and help develop and maintain body cells (Brufau et al., 2006, Ros and Mataix, 2006). Additionally, plants rich in monounsaturated fatty acids also contribute more vitamin E to the diet (Kim et al., 2017). The low content of saturated fatty acids is good for health, leading to a favorable decrease in serum total and LDL cholesterol and reducing HDL cholesterol fraction concentrations (Kim et al., 2017, Ros and Mataix, 2006).

*Minerals*. Minerals present in nuts belong to macro- and microelements. Every day humans need>22 mineral elements for maintenance of health and proper organ function. Hazelnuts, pistachio and almonds were characterized by high variability in the mineral contents (p ≤ 0.05) (Table 1) but macadamia and walnuts had the lowest (up to 960 mg/100 g). Potassium (464.5–1772.2 mg/100 g) and magnesium (197.0–502.5 mg/100 g) are the predominant minerals in the analyzed nuts. Pine, hazelnuts, pistachio and almonds are characterized by the highest content of potassium, whereas cashew, almonds and pine are rich in magnesium. Almonds and pistachio nuts are rich in calcium (Ca), cashew, walnuts, almonds and pine are characterized by high content of iron (Fe), cashew is rich in copper (Cu), cashew, pine and pecan are rich in zinc (Zn), and pine and hazelnuts are rich in manganese (Mn). Pecan, pine hazelnuts, almonds and walnuts have a lower content of sodium (Na). Additionally, some nuts contain selenium (Se, amounts given per 100 g), e.g. macadamia (13.1 µg), walnuts (6.8 µg) cashew (5.1 µg) or pine (3.5 µg) but in almonds and hazelnuts this microelement was not detected. These results are in agreement with those previously reported (Kumawat et al., 2017). Suliburska and Krejpcio (2014) analyzed the concentrations of Fe, Zn, Ca, and Mg in different types of nuts, in particular: Brazil nuts , cashews and hazelnuts . Brazil nuts were rich in Ca and Mg, cashews in Mg, and hazelnuts in Ca and Mg (Suliburska & Krejpcio, 2014).

The high content of potassium and lower sodium is important because they are responsible for maintenance of homeostasis, they regulate blood pressure and kidney function. Calcium is the most abundant mineral for humans, stored in bones and teeth, for properly functioning nervous and cardiovascular systems and muscles and additionally is a cofactor in numerous enzymatic reactions (Cosmulescu et al., 2013). Iron is an essential element for production of red blood cells responsible for transfer of oxygen from the lungs to tissues. Magnesium is a cofactor in>300 enzyme systems which regulate biochemical reactions in the body (Huskisson, Maggini, & Ruf, 2007), while micronutrients such as selenium are essential for the proper functioning of the human body. The consumption of eight Brazil nuts per day covers the RDA demand of selenium (55 µg) (Kumawat et al., 2017). Consumption of 100 g of hazelnuts supplies 10 % of calcium, about 55 % of phosphorus, 70 % of magnesium, about 13 % of potassium and 94 % of iron of RDA daily intake (Cosmulescu et al., 2013). Additionally, PCA analysis indicates that some minerals are associated with *in vitro* inhibitory enzyme activity by nuts. The content of Mn and Se is positively associated with intestinal α-glucosidase, AChE and BuChE, but Cu, Zn, Mg, Fe are associated with pancreatic α-amylase value (Fig. 1).

*Sugars*. The studied nuts contained from 0.9 (walnuts) to 2.5 g (pistachio) of sugars/100 g (Table 1). The most abundant sugar was sucrose, which constituted 94.1 to 100 % of all detected sugars (in the case of hazelnuts and pecan, respectively). Significantly lower concentrations of rhamnose and sorbitol were determined. In macadamia, pistachio, hazelnuts, and pine quantitated sorbitol was below 1.0 % of all sugars, while in other tested nuts it was not detected. Low content of sugars, especially the disaccharide sucrose presented a high negative Pearson correlation with the enzymes pancreatic α-amylase (r = -0.82) and intestinal α-glucosidase (r = 0.82). The enzymes present potent inhibition of metabolomic syndrome associated with high sugar content and improved the blood sugar content.

The results of Brufau et al. (2006) showed some similarities with the present results. The lowest content of sugars was detected in walnuts, and progressively increasing amounts were found in Brazil nuts, pecans, macadamia, almonds, pine, pistachios and, finally, cashews (Brufau et al., 2006). However, it well known that the nutritional composition of nuts, low in sugars but rich in protein, makes them a suitable food for inclusion in specialist diets intended for weight loss and control (Kim et al., 2017).

### Antidiabetic, anti-obesity and anticholinergic activity

Pancreatic α-amylase and intestinal α-glucosidase are two of the enzymes responsible for the breakdown of disaccharides and oligosaccharides into monosaccharides, which are compounds suitable for absorption. Pancreatic lipase is a key enzyme responsible for the hydrolysis of dietary fats into monoacylglycerols and free fatty acids and therefore exerts beneficial effects to reduce overweight and obesity in diabetic patients (Wojdyło, Nowicka, Turkiewicz, & Tkacz, 2021). The inhibition of these enzymes may be a good strategy to regulate the metabolic alterations related to type 2 diabetes and hyperglycemia (Kim et al., 2017, Noguera-Artiaga et al., 2019). The inhibition of the enzymes pancreatic α-amylase, intestinal α-glucosidase, and pancreatic lipase by different nuts was assessed and reported in Table 1. It was observed that most of the analyzed nuts present weak activity against pancreatic α-amylase. Pistachio and cashew nuts showed activity < 30 % (IC_50_ = 318.9 and 318.9, respectively) while other nuts such as pine (>43 %, IC_50_ = 262.6) and pecan (>60 %, IC_50_ = 148.9) showed the highest inhibitory activity at 1 mg/mL. Compared to the positive control acarbose showed an IC_50_ value of 0.1 mg/mL and 74 %.

Pearson’s coefficient (Fig. 2) showed positive correlations between the pancreatic α-amylase and flavonols (r = 0.70, p > 0.05), a weak correlation with tocopherol (r = 0.32, p > 0.05) and a negative correlation with tocotrienol content (r = -0.50, p > 0.05). Pancreatic α-amylase and intestinal α-glucosidase presented a weak negative correlation with sugars (r = -0.27 and −0.20, respectively; p > 0.05).

In the same trend, all the samples presented the same level of activity against pancreatic lipase. Pecan, pistachio and walnuts presented higher activity, i.e. 69, 68 and 57 %, respectively. The lowest potential towards pancreatic lipase was presented by almonds and pine nuts (36 and 38 %, respectively). Compared to the positive control acarbose showed a percentage of 73 % and an IC_50_ value of 0.1 mg/mL. Pancreatic lipase was positively associated with phenolic acid (r = 0.64, p < 0.05) and tocopherols (r = 0.57, p < 0.05), and showed a weak correlation with sugars, total fat, SFAs, MUFAs and PUFAs (r = 0.01, 0.27, 0.02, −0.23 and 0.22, respectively, p > 0.05).

Interestingly, the samples displayed an inhibitory effect against intestinal α-glucosidase (Table 2), with % of inhibition>93 % for 1 mg/mL. The highest activity of nuts against intestinal α-glucosidase was shown by pecan (99 % inhibition and IC_50_ = 13.9 at 1 mg/mL) followed by almonds (93 % inhibition and IC_50_ = 18.5 at 1 mg/mL, respectively). The acarbose control showed an IC_50_ value of 2.6 mg/mL. Triterpene presented negative Pearson correlations with intestinal α-glucosidase (r = -0.46, p > 0.05) and with flavanols (r = -0.60, p > 0.05).

Recently it has been reported that regular consumption of nuts is associated with an improvement in the conditions of health, e.g. type-2 diabetes and metabolic syndrome (Kim et al., 2017). Results obtained by Pino Ramos et al. (2019) show some similarities with the present results. Pistachio consumption improved glycemic status in subjects with type-2 diabetes mellitus (Kim et al., 2017). Also consuming almonds improved glucose control in subjects with impaired fasting glucose (for Kim et al., 2017) and pre-diabetes. Similarly, walnuts (30 g/day) added to the low-fat diet of type-2 diabetic patients improved their blood lipid profiles (Rajaram & Sabaté, 2006). However, in the future more studies are needed for other nuts to support these observations.

One of the potential therapeutic strategies in Alzheimer and Parkinson diseases is to increase the cholinergic levels in the brain by inhibiting the biological activity of acetylcholinesterase (AChE) and butylcholinesterase (BuChE). Therefore, still active research is being conducted to find new acetylcholine inhibitors used to limit their degradation (Amat-Ur-rasool et al., 2020).

Therefore, in this research additionally the nuts were evaluated for their inhibitory effect towards the enzymes AChE and BuChE. The most active extracts from nuts against AChE were those from pecan and pine nuts (29 and 27 %), but the other samples showed weak activity below 20 %. Hazelnuts, pistachio, almonds, and cashew are nuts present marginal inhibition of BuChE. The remaining nuts (pecan, pine and macadamia) show slightly higher inhibition potential. Fig. 2 presents the Pearson correlation coefficient results. BuChE negatively correlated with minerals and total fat (r = -0.76 and −0.78, respectively, p < 0.05). For BuChE and AChE activity the influence presented by biologically active compounds such as phenolic acid (r = -0.47 and −0.36), flavonols (r = 0.29 and 0.47), tocopherols (r = -0.34 and 0.27), and triterpene (r = 0.37 and 0.56), respectively, was in each case non-significant, p > 0.05. Reports of anti-AChE or anti-BuChE activities from extracts of nuts are absent from the literature. Leaf extract polyphenols of *L. inermis* (Henna) and *E. globulus* (Eucalyptus) were the most potent anti-AChE and anti-BuChE inhibitor (Amat-Ur-rasool et al., 2020).

## Conclusion

Eight different nut samples, namely pecan, pine, hazelnuts, pistachio, almonds, cashew, walnuts, and macadamia, were studied to assess the content of bioactive compounds comprising polyphenols, tocochromanols, and triterpene, and nutritional ingredients constituting fat, fatty acids, minerals, and sugars. In general, the polyphenol content was low, tocopherol homologs were the predominant tocochromanols, and nuts were rich in triterpene compounds, which were responsible for moderate *in vitro* enzyme inhibitory. Nuts, especially hazelnuts and almonds, are excellent sources of natural vitamin E. For the first time in nuts triterpene compounds were quantified. Pine nuts and hazelnuts are a source of 11 triterpenes, of which oleanolic and pomolic acids were the dominant compounds. Nuts are low in sodium, iron, copper, zinc, and manganese but a good source of potassium and magnesium. Some nuts, i.e. macadamia, walnuts, and cashew, turned out to be a good source of selenium. Among all tested nuts, hazelnuts, pistachio, and almonds had the highest content of minerals compared to popular consumer fruits and vegetables. According to the results obtained from this study, fat accounts for almost 30–45 % of the weight in nuts; however, the fat of nuts is primarily composed of monounsaturated fatty acids, mainly predominant in hazelnuts, macadamia, almonds, cashew, pecan, and pistachio.

In relation to the above, all nuts showed high activity in inhibiting intestinal α-glucosidase (>90 %), but there was lower ability to inhibit activity of the enzymes pancreatic α-amylase and pancreatic lipase. The activity in inhibiting acetyl- and butylcholinesterase was up to 30 %.

As presented above, all nuts contain a wide array of compounds that improve the nutritional value; therefore they should be one of the important components of the daily human diet rich in functional foods. This is extremely important in today’s busy lifestyles, because nuts are a convenient and tasty snack that contributes to a healthy lifestyle.

## Ethic statement

Research did not include any human subjects and animal experiments.

## CRediT authorship contribution statement

**Aneta Wojdyło:** Methodology, Resources, Supervision, Conceptualization, Data curation, Formal analysis, Visualization, Writing – original draft, Writing – review & editing. **Igor Piotr Turkiewicz:** Formal analysis, Methodology, Data curation. **Karolina Tkacz:** Formal analysis, Methodology, Data curation. **Paulina Nowicka:** Methodology, Data curation, Formal analysis. **Łukasz Bobak:** Methodology, Formal analysis, Data curation.

## Declaration of Competing Interest

The authors declare that they have no known competing financial interests or personal relationships that could have appeared to influence the work reported in this paper.
